# A critical path to producing high quality, reproducible data from quantitative western blot experiments

**DOI:** 10.1038/s41598-022-22294-x

**Published:** 2022-10-20

**Authors:** Sean C. Taylor, Luciana K. Rosselli-Murai, Bélinda Crobeddu, Isabelle Plante

**Affiliations:** 1 GENSCRIPT USA INC, 860 Centennial Ave., Piscataway, NJ 08854 USA; 2 Institut National de la Recherche Scientifique - Armand-Frappier Santé Biotechnologie, 531 Boul. des Prairies, Édifice 18, Laval, QC H7V 1B7 Canada

**Keywords:** Enzyme mechanisms, Proteins, Biomarkers, Medical research

## Abstract

Western blotting experiments were initially performed to detect a target protein in a complex biological sample and more recently, to measure relative protein abundance. Chemiluminescence coupled with film-based detection was traditionally the gold standard for western blotting but accurate and reproducible quantification has been a major challenge from this methodology. The development of sensitive, camera-based detection technologies coupled with an updated technical approach permits the production of reproducible, quantitative data. Fluorescence reagent and detection solutions are the latest innovation in western blotting but there remains questions and debate concerning their relative sensitivity and dynamic range versus chemiluminescence. A methodology to optimize and produce excellent, quantitative western blot results with rigorous data analysis from membranes probed with both fluorescent and chemiluminescent antibodies is described. The data reveal when and how to apply these detection methods to achieve reproducible data with a stepwise approach to data processing for quantitative analysis.

## Introduction

Western blotting was first introduced in 1979 as a means of detecting a target protein in a sample with antibodies and detection using film^1,2^. Over the following two decades, as reagent and antibody technologies evolved, scientists attempted to produce quantitative results initially by scintillation counting and film densitometry^3,4^. Since the linear quantitative range of film is only about an order of magnitude and very challenging to assess saturation by eye, the results even between technical replicates were difficult to reproduce^5^. During this time, the first camera-based imaging systems were introduced which enabled automated detection over a much broader linear dynamic range of about three to four orders of magnitude^6^. This permitted the generation of semi-quantitative data from chemiluminescence. However, the original qualitative techniques and assumptions that have been passed down through generations of lab members were not formally updated to permit the production of semi-quantitative western blot data thus giving poor reproducibility and misleading interpretations^7–9^. The associated retractions from high profile articles^10–13^ support the unfounded reputation of western blotting as a difficult, frustrating and non-reproducible technique that is semi-quantitative at best. Recent articles have dissected and contrasted the old practices passed down from the early days of western blotting to offer revised methodologies that take full advantage of the currently available state-of-the-art instruments and reagents to achieve accurate, precise and reproducible data^14–16^. These highly viewed and well cited articles support the need for updated guidance in producing quantitative western blot data. However, a complete and concise guide to the workflow highlighting the importance of reagent selection, data workup and analysis remains elusive.

With limiting sample availability to assess components of microdissected tissue, sorted cell subpopulations, exosomes and primary cells cultured from small tissue specimens, the requirement to maximize quantity while preserving the quality of data is paramount^17,18^. Multiplexed target detection from chemiluminescent western blots is feasible when working with highly specific antibodies against targets that differ in molecular weight by at least five to ten kilodaltons (kDa). However, chemiluminescence is not amenable to multi-target detection when using antibodies with low specificity or for proteins that are very close in molecular weight with overlapping signals. This often requires regeneration of the membrane by stripping off the antibodies and reprobing^19^. Unfortunately, the stripping process is time consuming and often removes variable amounts of protein and antibodies to potentially give low quality, variable and, in some cases, artefactual data^20^. Furthermore, the byproducts of horseradish peroxidase (HRP) induced chemiluminescence are free radicals which can inactivate the HRP itself (giving a visible yellow/brown color) and irreversibly damage the antibodies, proteins and membrane itself prohibiting stripping and reprobing^21^.

Fluorescent western blotting permits the interrogation of multiple targets from the same sample while avoiding the stripping/reprobing process with several advantages (Table 1).

**Table 1:** Key comparative factors between chemiluminescence and fluorescence detection.

Key Factors	Chemiluminescence	Fluorescence
Price	Low	High
Sensitivity	High	High
Multiplexed target detection	No	Yes
Signal stability	No	Yes

Although fluorescence detection is more expensive than chemiluminesence due to the increased cost and concentration of secondary antibodies, the multiplexed data can offer significant improvements^22,23^. Furthermore, the simultaneous detection of multiple proteins greatly reduces the sample requirements that typically pose the greatest expense in most experiments. However, the question around the comparative sensitivity, linear dynamic range and reproducibility of fluorescence versus chemiluminescence detection has generated conflicting views^24^. This has been exacerbated by the vast number of available fluorescent secondary antibodies and general confusion around their practical application to a typical western blot protocol.

There has been limited published data directly comparing fluorescence with chemiluminescence that partially explains the confusion surrounding how and when to apply these different detection methods for quantitative western blotting. Thereby, this project aims to provide a direct comparison of data derived from identical blots using a combination of fluorescent and chemiluminescent detection antibodies. Furthermore, a simple and concise, stepwise methodology for producing excellent and reproducible quantitative western blot data with associated data analysis is described.

## Results

### Direct comparison between fluorescence and chemiluminescence detection and analysis

In order to directly compare the sensitivity, reproducibility and linear dynamic range between chemiluminescence and fluorescence detection, three, identical membranes were co-incubated with the primary antibodies against phospho-β-catenin, β-catenin, and α-tubulin (Table 2). The membranes were split such that each set was incubated with the same mixture of fluorescent secondary conjugates (Table 2) and either goat-anti-rabbit or goat-anti-mouse secondary HRP conjugates for chemiluminescence detection. Thus permitting the detection of all three target proteins from the same membrane with fluorescence along with either phospho-β-catenin (goat-anti-rabbit-HRP) or β-catenin and α-tubulin (goat-anti-mouse-HRP) with chemiluminescence.

**Table 2:** Primary and secondary antibodies.

Antibody	Supplier	Catalogue number	Dilution factor
α-Tubulin, mouse	GenScript	A01410	1/2000
phospho-β-catenin (Ser675), rabbit	Cell signaling	4176	1/1000
β-catenin, mouse	Cell signaling	2677	1/5000
Goat-anti-rabbit-HRP	GenScript	A00098	1/2000
Goat-anti-mouse-HRP	GenScript	A00160	1/5000
Goat-anti-mouse-alexa 647	Cell signaling	4410S	1/1000
Goat-anti-rabbit-dylight 488	ThermoFisher	35552	1/1000

### Fluorescence detection and analysis

**Figure 1:**
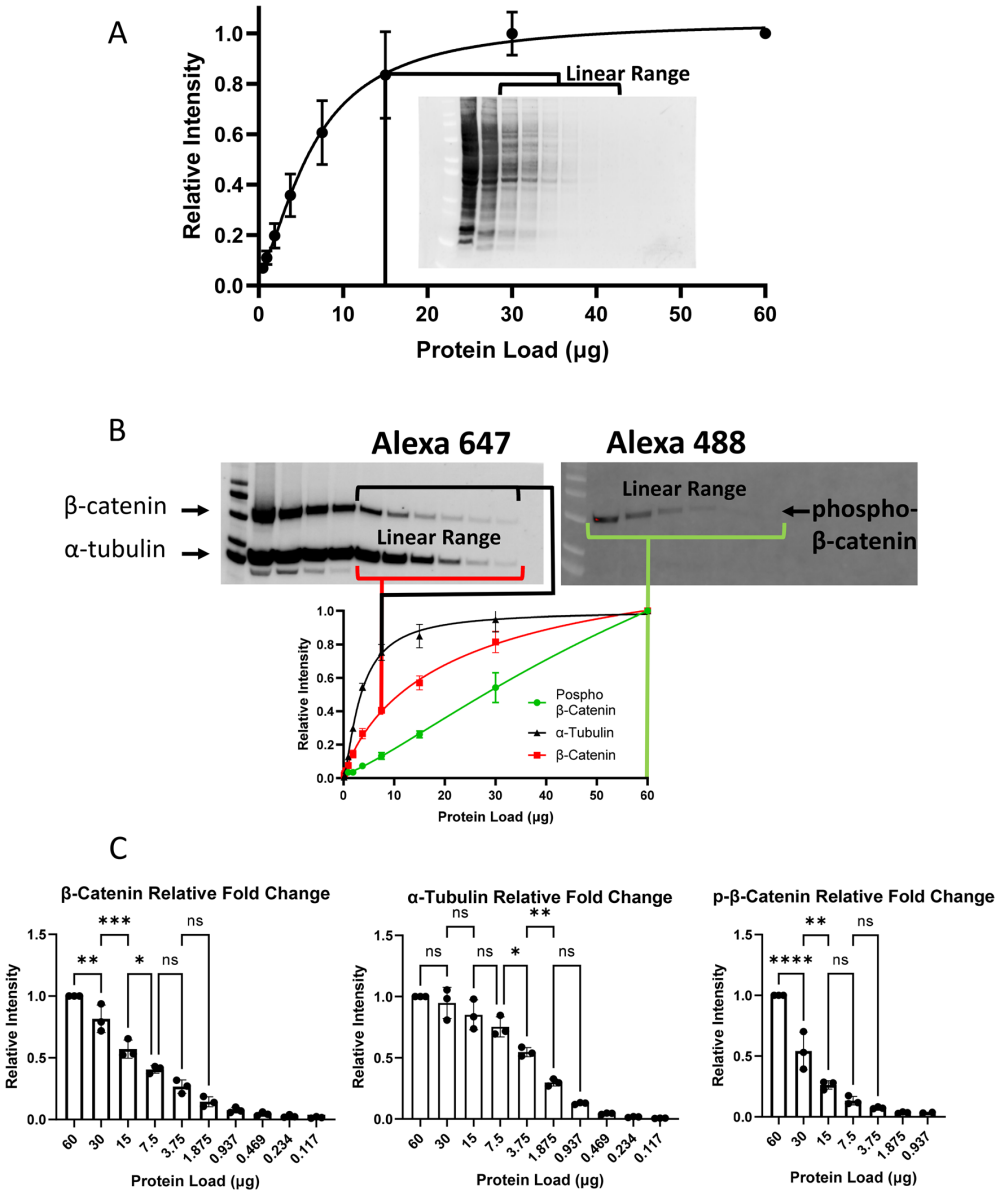
Fluorescence data analysis. Three separate blots were independently generated from the same 1:2 sample dilution series of an equalized pool of cell lysates starting from approximately 60 µg total protein loading. The membranes were probed with a mixture of three primary antibodies against β-catenin, phospho-β-catenin and α-tubulin followed by a solution of mixed fluorescent and chemiluminescent secondaries (see "Methods" section and Table 2). (**A**) Representative image of the total protein fluorescent signal from each membrane and associated plot of the average relative fluorescence intensity for each dilution (error bars represent the SEM from the average of three, separate experiments). (**B**) Representative image of the fluorescent signal from each protein target and associated plot of the average relative fluorescence intensity versus total protein loading amount (error bars represent the standard error of the mean (SEM) from the average of three, separate experiments). The bracketed region for each target protein represents the linear dynamic range of quantitation. (**C**) Bar charts depicting the average, relative signal intensity versus total protein loading amount for the three experiments (error bars represent the standard deviation (SD) from the average of three, separate experiments). Data analysis was performed according to Fig. 4, column 6 (Relative Normalized Ratio (RNR)). The statistical difference between each level of protein load is shown where ****p-value < 0.0001, ***p-value < 0.0002, **p-value < 0.0021, *p-value < 0.0332; *ns* non-significant. Full blot images for (**A**) are provided in the supplemental data (Supplemental Fig. 1).

The linear dynamic range of different protein targets was assessed using fluorescent conjugated antibodies (Table 2 and Fig. 1). Fluorescent conjugated secondary antibodies with large spectral resolution have several advantages including the simultaneous quantification of multiple proteins from the same membrane (Table 1)^23,25^. This is particularly advantageous for proteins that resolve at the same molecular weight as is the case for phospho-β-catenin and β-catenin. The selection of primary antibodies from different species is required for the simultaneous incubation of fluorescent secondary antibodies as was the case for this work (Table 2). Total protein was measured on the transferred membrane prior to blotting as a quality control procedure to confirm a well-defined and consistent band pattern in each lane and a decreasing signal intensity reflective of the 1:2 dilution series applied to this experiment within the linear range (Fig. 1A). The signals from each target were well resolved within the same membrane (Fig. 1B). A standard curve of relative fluorescent intensity against total protein load revealed the linear dynamic range (Fig. 1B—chart: bracketed region of dilutions for each target highlighting dynamic range of protein load) and plateau region for quantification of each target (indicating overloaded protein). As expected, the more abundant proteins (β-catenin and α-tubulin) reached the point of membrane saturation (start of the plateau region, around 10 µg) at a lower level of total protein loading than the lower abundant protein (p-β-catenin) which gave a linear signal to 60 µg protein loading (Fig. 1B—chart). The statistical difference between the averages of three separate experiments for each 1:2 dilution was tested and for all three proteins, there was a statistically significant difference between at least two of the 1:2 dilutions within the linear range (Fig. 1C).

### Chemiluminescence detection and analysis

**Figure 2:**
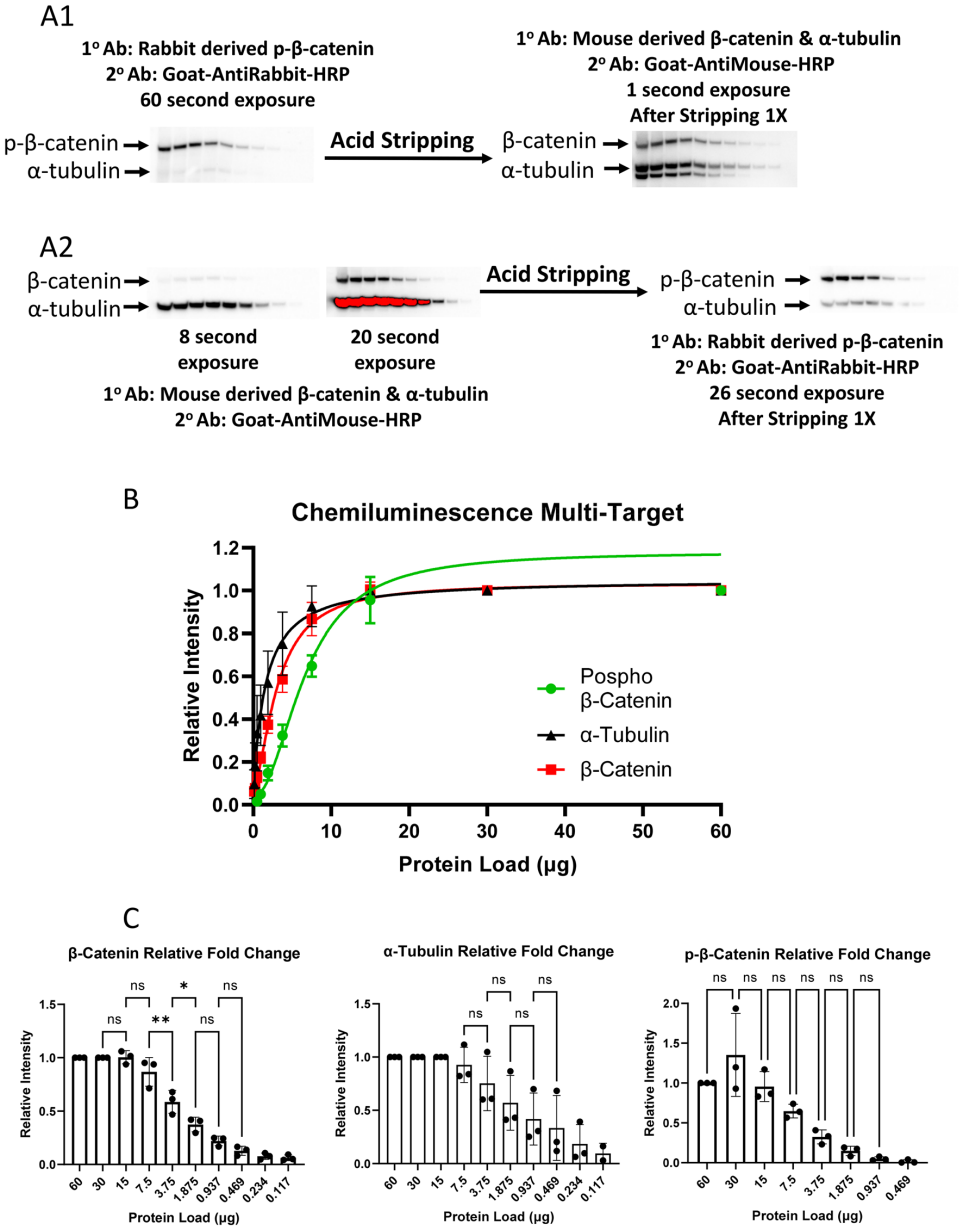
Chemiluminescence data analysis. The identical three blots assessed by fluorescence (Fig. 1) were also analyzed by chemiluminescence. The membranes were probed with a mixture of three primary antibodies against β-catenin, phospho-β-catenin (p-β-catenin) and α-tubulin followed by a solution of mixed fluorescent and chemiluminescent secondaries (see "Methods" section). (**A1**) Chemiluminescent signal from p-β-catenin followed by membrane stripping and probing for β-catenin and α-tubulin. (**A2**) Chemiluminescent signal from β-catenin and α-tubulin followed by membrane stripping and probing for p-β-catenin. (**B**) Plot of the average relative density versus protein load from the combination of both unstripped and stripped membranes (error bars represent the SEM from the average of three, separate experiments). (**C**). Bar charts depicting the average, relative signal intensity versus protein load for the three experiments from both unstripped and stripped membranes (error bars represent the standard deviation (SD) from the average of three, separate experiments). The statistical difference between each level of protein load is shown where: **p-value < 0.0021; *p-value < 0.0332; *ns* non-significant. Full blot images for (**A**) are provided in the supplemental data (Supplemental Fig. 2).

Since phospho-β-catenin and β-catenin resolve at the same molecular weight even when using a gradient SDS-PAGE gel, their signals cannot be resolved and quantified from the same membrane by chemiluminescence with HRP-conjugated secondary antibodies. Thus, for the replicate experiments, the fluorescent secondary antibodies were co-incubated with either goat-anti-rabbit or goat-anti-mouse HRP conjugates for chemiluminescence detection of phospho-β-catenin and β-catenin respectively on separate membranes. In order to detect the opposing target on the same membrane, stripping and reprobing was required (Fig. 2A). Hence, for the membranes where goat-anti-rabbit HRP was initially used, phospho-β-catenin was well resolved with good sensitivity and linearity of signal (Fig. 2A1). Following an overnight stripping protocol (see "Methods" section) and re-incubation with the goat-anti-mouse HRP secondary conjugate, the signals for both β-catenin and α-tubulin were well resolved (Fig. 2A1). For the membranes initially co-incubated with goat-anti-mouse HRP and fluorescent secondary conjugates, α-tubulin and β-catenin were well detected (Fig. 2A2). After stripping overnight, the membranes were incubated with the goat-anti-rabbit HRP for signal for detection of p-β-catenin (Fig. 2A2).

Since the membranes used for chemiluminescent detection were identical to those used for fluorescence the total protein signal was conserved (Fig. 1A). For each target, the relative mean densitometric data from the same three membranes used for fluorescence (either pre or post stripping) were plotted to produce a standard curve of chemiluminescence density versus total protein loading amount (Fig. 2B). The linear range for the three targets (particularly for p-β-catenin) was truncated in comparison to the fluorescent data (compare Figs. 1B and 2B). Also, the precision and accuracy between the replicate data was higher with the fluorescent secondary antibodies (compare error bars between Figs. 1B with 2B at each dilution for each target). With chemiluminescence, only for β-catenin was there a statistically significant difference between two of the serial dilutions in the linear range (Fig. 2C) whereas with fluorescence, a statistically significant difference between at least two dilutions was measured for all three targets (Fig. 1C).

## Discussion

**Figure 3:**
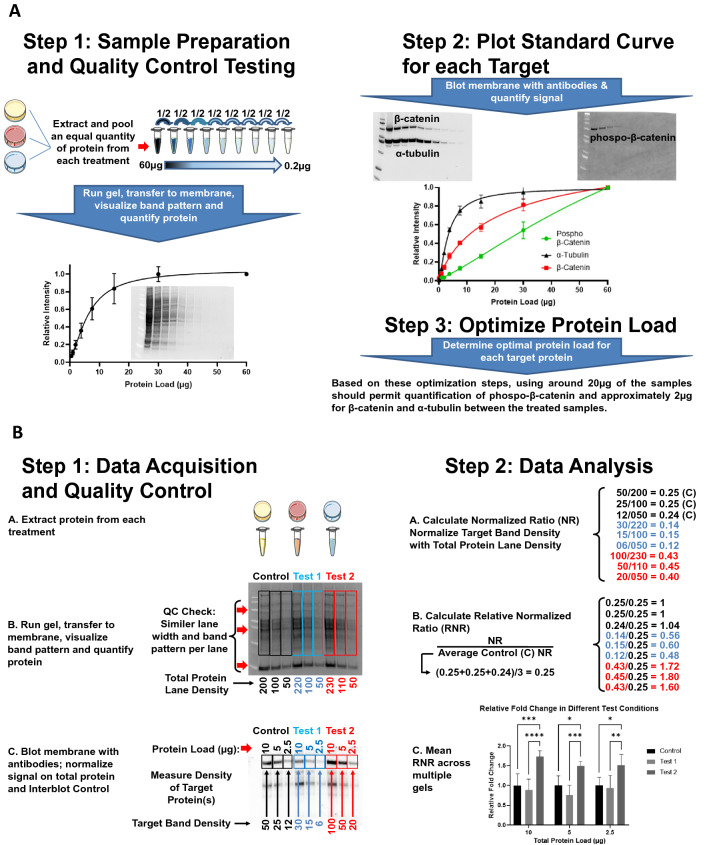
Quantitative western blot workflow summary. (**A**) Optimization of the assay for the specific set of samples using a specific target. Step 1: Sample Preparation and Quality Control Testing. Produce a pooled lysate from all treatments to a maximum initial concentration of 4 µg/µl and produce a standard curve by diluting serially by a factor of 1:2 for seven dilutions loading 20 µl per well on an SDS-PAGE gel. After gel-based separation, transfer to membrane using a consistent transfer apparatus and assess the total protein transferred (see "Methods"). Step 2: Plot Standard Curve for each Target. Probe the membrane with each antibody. Plot the relative density of each protein versus protein load to generate a standard curve. Step 3: Optimize Protein Load. Determine the optimal total protein load by choosing the protein load corresponding to the middle of the standard curve linear range for each target. This offers the best chance of achieving target-specific quantitative data from the individual samples. (**B**) Quantification of Target Expression. Step 1: Data Acquisition and Quality Control. Assure that the total protein loaded per lane is within the linear dynamic range of the target protein for accurate normalization and sample quality verification. Step 2: Data Analysis. Use a similar methodology as qPCR^40^ to work up the data from western blotting to assure the results reflect the tested experimental conditions. The blot images from this figure are strictly to depict the western blot workflow, associated quality controls and data analysis steps with contrived data to clarify the data analysis steps.

**Figure 4:**
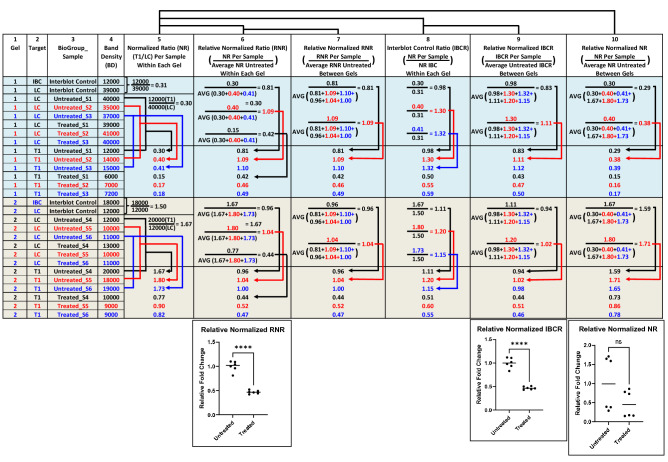
Western blotting data analysis workflow (example using contrived data). The data analysis workflow can be tabulated in EXCEL with formula propagation (see supplemental WB Data Analysis EXCEL Spreadsheet). The columns are partitioned to incorporate the critical data components and calculations: (1) Gel number. (2) Target: Interblot Control (IBC): The IBC is recommended to be an equalized pooled sample from the positive experimental samples from each biological group. LC: Loading Control: The loading control used for normalization. Either the lane density of total protein or a specific loading control protein that does not change in expression between biological groups. T1: The target protein tested for expression differences between biological groups. (3) BioGroup_Sample: The individual samples within each biological group (i.e.: the tested treatment/condition). (4) Band Density: The density/volume of a given protein band as revealed with an imaging system from a fluorescence or chemiluminescence signal. (5) Normalized Ratio (NR): The density ratio of the target protein versus the loading control within each gel lane (sample). (6) Relative Normalized (RNR): The NR per sample divided by the average NR for the untreated (control) samples within each gel. (7) Relative Normalized RNR: The RNR per sample divided by the average RNR for untreated (control) samples spanning a given experiment (between gels). (8) Interblot Control (IBC) Ratio (IBCR): NR per sample divided by the NR of the IBC within each gel (Controls for any inter gel/blot variability in sample loading, transfer efficiency and other sources of experimental variability when running multiple gels for the same target). The IBCR is an essential control when the number of samples for a given experiment exceed the number of available wells of a single gel. (9) Relative Normalized IBCR: The IBCR per sample divided by the average IBCR for untreated (control) samples spanning a given experiment (between gels). (10) Relative Normalized NR: The NR per sample divided by the average NR for untreated (control) samples spanning a given experiment (between gels). The charts demonstrate the statistical significance between the same set of treated and untreated samples based on the different calculation methodologies. ****p-value < 0.0001, *ns* non-significant.

In order to achieve semi-quantitative data through the densitometric analysis of western blots, an evolution in the experimental design (Figs. 1, 2 and 3), data validation (Figs. 3 and 4), verification (Fig. 3) and associated analysis (Fig. 4) procedures is required to assure high quality, reproducible results. Three of the most critical considerations include the total protein load, sample quality and the approach to normalization^16,26–28^.

Since protein expression analysis is highly sensitive to the experimental conditions posed on the cells, tissue or specimens, it is impossible to estimate the amount of a given lysate to load for SDS-PAGE to assure that the target protein will be quantifiable after transfer to the membrane. Therefore, the linear quantitative range must be determined from a standard curve generated from a pool of protein lysates from each biological group (i.e.: treatments) representing the average expression of the individual experimental samples (Figs. 1B and 3A)^14,15^.

For each transferred membrane, the sample quality can be assessed by comparing the total protein band pattern and density between lanes visualized and measured using a fluorescent dye that does not influence the downstream antibody incubations (Figs. 1A and 3A). Total or partial lane density can also be used for western blot normalization (Fig. 3B) eliminating the requirement for traditional loading control proteins such as B-actin, α-tubulin or GAPDH that are all typically overloaded on membranes due to their high expression levels^14,15^.

### Fluorescent versus chemiluminesent antibodies: myths and truths

Fluorescent secondary antibodies are generally viewed as more quantitative and sensitive than chemiluminescent secondaries^22–24^. One clear advantage is the ability to use fluorescent secondary antibodies of different emission wavelengths for the simultaneous detection and quantification of multiple proteins including those that resolve within about 5 kDa as was the case for β-catenin and p-β-catenin (Fig. 1B)^23^. This precludes the requirement to either strip and reprobe or to use multiple blots when analyzing different proteins, as required for chemiluminescence (Fig. 2A), which introduces technical variability and the potential for reduced statistical significance between biological groups (compare Figs. 1C with 2C)^29^.

The design of this experiment permitted the direct comparison of fluorescent and chemiluminescent data derived from the same membrane for each protein. For both detection methods, the linear quantitative range was similar for β-catenin and α-tubulin (compare Fig. 1B with 2B). Thus, the upper and lower limits of quantification were similar for both detection methods. However, for p-β-catenin, the linear range was much broader using fluorescent detection. Also, each target exhibited less variability between each 1:2 dilution for the fluorescent standard curve as opposed to chemiluminescence (compare error bars between Fig. 1B with 2B). This generally supports a higher level of precision when using fluorescent secondary antibodies leading to more statistically significant data between biological groups. This is evidenced by comparing Figs. 1C with 2C where there were more statistically significant differences were observed between the sample dilutions for all targets with fluorescence detection.

The higher variability for chemiluminescence in these comparative experiments is likely attributed mainly to the increased technical variability from stripping and reprobing the membranes coupled with the transient nature of signal production with chemiluminescent detection. Membrane stripping procedures vary widely in buffer compositions, incubation times and temperatures. This can result in variability caused by reprobing the same stripped membrane from either loss of protein, inadequate removal of antibodies and excessive HRP-induced, free-radical generation^21,29,30^. For previous work, the interblot variability for chemiluminescence signals from fresh, unstripped membranes was comparatively lower^14,15^ but fluorescent detection remains advantageous (Table1). Since camera-based imaging systems have evolved to detect both chemiluminescent and fluorescent signals, multiple target proteins can now be detected from the same, unstripped membrane using a combination of fluorescent and chemiluminescent secondary antibodies to minimize variability and maximize the data from a single sample^23^.

### The importance of the linear quantitative range for quantification of both fluorescence and chemiluminescence data analysis

For these experiments and samples, 10 µg of total protein load represents the upper limit of quantification (ULOQ) and the beginning of the plateau region of the standard curve for both α-tubulin and β-catenin where the membrane is protein-saturated (Figs. 1B and 2B). Results from protein loading greater than the ULOQ do not reflect the true expression differences between samples and in fact would show almost no difference^14,15^. Whereas within the linear range, the densitometric differences were statistically significant for all proteins with fluorescence (Fig. 1C) and for β-catenin with chemiluminescence (Fig. 2C). In order to ensure densitometric data that reflects the true expression differences between samples, it is best to use a dilution factor that approximately aligns to the middle of the linear range for each protein target (Fig. 3).

The linear quantitative dynamic range can be achieved by producing an equalized pool of individual samples from each biological group including control that can be serially diluted 1:2 from a starting total protein loading amount of about 60ug for at least six dilutions (ie: 60, 30, 15, 7.5, 3.75, 1.875 µg; Fig. 3A)^15^. The data from these particular experiments and samples indicate that in order to be quantitative with fluorescent secondary antibodies, a protein load of approximately 2 µg would be required for β-catenin and α-tubulin whereas for p-β-catenin it is necessary to load about 20 µg (Figs. 1B and 3A). Thus, for different proteins with varying expression levels, large differences in total protein load that exceed an order of magnitude may be required to achieve data that reflect the true/real changes in protein expression. Furthermore, for total versus phosphorylated proteins, it may be necessary to produce the data from different membranes to assure the total protein is not overloaded in the quantitative linear loading range of the phosphorylated counterpart.

### Different lysis buffers can give different results

The solubility of individual proteins can vary dependent on several factors including: (1) the number of exposed hydrophobic amino acids and post-translational modifications, (2) tertiary and quaternary structure, (3) the surrounding environment and/or cellular localization, (4) whether they are hetero or homo complexed^31–33^. Thus, the choice of lysis buffer can affect the solubility of individual proteins and hence their detection^34^. Even heating a sample at 95 °C in Laemli buffer, as is normally performed for SDS-PAGE protein loading, can affect the detection of certain protein subclasses in western blotting^35^.

### Steps to assure quantitative data from western blots

In order to achieve quantitative densitometric data from western blots, determination of the appropriate total protein load assures optimal dose-dependent responses per target (Figs. 1A and 3A)^15,30^. Since there is an approximate one million-fold difference between high and low expressed proteins in a given tissue or cell lysate, loading the same amount of total protein per lane there will invariably give targets that are over and underloaded^36^. This can ultimately result in densitometric data that are outside the linear dynamic range of quantification for each target and results that may not reflect the experimental conditions^15,16,29,30^.

Loading controls to normalize the data between lanes and blots have become a highly debated subject over the past decade^37,38^. Traditionally, labs have used housekeeping proteins such as α-tubulin, β-actin or GAPDH to normalize western blots based on the assumption that these proteins are stably expressed between samples and across experimental conditions. However, there is now substantial evidence cautioning the use of such proteins for normalization because of their very high abundance, the impact of treatment on their expression and thus lack of consistency between samples^15,37,38^. The use of total protein membrane stains is now becoming the method of choice to assure appropriate normalization between samples (see "Methods" section and Figs. 1A and 3B). An additional major advantage to using total protein for normalization is that the resulting image of the total transferred protein on the membrane offers a quality control checkpoint for the samples themselves by comparing the band pattern, lane width and density between each lane (Fig. 3B). In order to assure excellent, normalized results, both the target protein and loading control must be within their respective linear dynamic ranges from the same membrane (Figs. 1, 2, 3). Although, this does add complexity to a western blot experiment in the validation of each target protein and the loading control with the requirement to produce a preliminary standard curve, it assures the production of high quality, reproducible data for quantification (Fig. 3).

### Data Analysis Workflow

There are no strict norms for western blot data analysis and as a result there is no consistency in this process between and even within labs^5,39^. This can lead to data that are difficult or impossible to reproduce entirely stemming from analysis inconsistencies. The trend towards normalization and quality control of western blots from the total transferred protein image of the membrane^14,15^ necessitates an experimental design that accommodates the acquisition of two, different images from the membrane. The initial “Total Protein” image is typically acquired immediately after the proteins have been membrane transferred from the SDS-PAGE gel using a total protein fluorescent dye as described in the "Methods" section (Fig. 3B). After incubating the membrane with the primary and secondary antibodies, the “Target Protein” image is taken using either fluorescence or chemiluminescence (Fig. 3B). Since each image is captured at different times, instrument settings and photon emission sources, the resulting normalized densitometric ratios (i.e.: [Target Protein − Lane X]/[Total Protein − Lane X]) can be significantly different between blots (see Fig. 4, column 10). The main sources of variability include transfer efficiency, exposure time and antibody activity potentially giving non-significant data between the combined results for the control and treatment biological groups that are entirely consequent to the data analysis approach (Fig. 4—Column 10 data and chart entitled “Relative Normalized NR”). Therefore, high quality data is best achieved by first producing normalized expression data within each blot followed by calculating the mean, normalized expression between blots (Fig. 4—Column 6 data and chart entitled “Relative Normalized RNR”). However, most western blot experiments involve samples sets that exceed the available number of lanes in a single gel. It is therefore good practice to reserve at least one or two lanes on each gel for an interblot control (IBC) sample loaded at a dilution for which the target protein signal is well within the linear range^15^. This permits the calculation of relative expression based on the normalized sample ratio to that of the IBC within each gel (ie: ([Target Protein − Lane X]/[Total Protein − Lane X])/([Target Protein − IBC Sample]/[Total Protein − IBC Sample])) (Fig. 4—Column 9 data and chart entitled “Relative Normalized GCR (RNG)”). Figure 4 is also available as an EXCEL Spreadsheet template (Supplemental Data).

## Conclusion

Quantitative western blotting is perceived to be one of the most challenging and frustrating techniques to achieve consistent and quantitative data. The root cause of poor data is typically associated with the application of the techniques and methodologies that were used decades ago that have been passed down through generations of scientists. By using an updated workflow (Fig. 3) and data analysis methodology (Fig. 4), consistent, reproducible and quantitative data can be achieved.

## Methods

### Tissue homogenization and cell lysates

Epithelial Breast Cancer Cells (T47-D cells, ATCC) were plated and grown Roswell Park Memorial Institute medium (RPMI) without phenol red, supplemented with 10% of fetal bovine serum, 0.2 Units/ml of bovine insulin, 1 mM of HEPES, 2.4 μg/mL of glucose and 1 mM of sodium pyruvate (all from ThermoFisher Canada, Mississauga, ON). Cells were kept in a humidified incubator with 5% CO_2_ at 37 °C until they reached ≈90% confluence. The cells were rinsed twice with ice-cold PBS and then harvested in 500 µl of lysis buffer added directly to the plate with scraping. The homogenates were transferred to 1.5 ml microtubes and incubated on ice for 15 min. The cell homogenates were then centrifuged for 5 min at 1000 g at 4 °C. The resulting supernatants were aliquoted and kept at -80 °C.

Lysate protein concentrations were measured using the Bicinchoninic acid protein assay reagent kit (Thermo scientific). Before being used for western blotting, the lysates were mixed with a solution containing 950 µL 4 × LDS Sample Buffer (GenScript – M00676) with 50µL of 8 M DTT to a working concentration of 3 to 4 µg/µL of total protein.

### Antibodies

Table 2.

### Gels and western blot reagents and equipment

4–12% SurePAGE, Bis–Tris Gel, 12 well, 80 µl (GenScript – M00653) were used for protein separation. MES Buffer Powder packs (GenScript – M00677) were reconstituted in one liter of ddH_2_O for gel separation in GenBox Mini Tank (GenScript – L00780).

The Complete eBlot L1 Transfer sandwich (PVDF) (GenScript – L00727) was used in conjunction with the eBlot L1 Fast Wet Transfer System (GenScript – L00686) using the preset “Standard” setting for all transfers requiring 16 min per membrane.

PVDF membranes were activated in 100% Ethanol with gentle shaking for approximately five to ten minutes and then equilibrated in eBlot L1 PVDF Membrane Equilibration Buffer (1X) for about 2–5 min prior to transfer. After protein separation, the gels were incubated in water for about two minutes prior to transfer.

### Confirming transfer by membrane staining using sypro ruby protein blot stain (Thermo-Fisher—s11791)

Post transfer, the membranes for the cell lysates underwent the following procedure:

1. Rinsed three times in ddH_2_O for about one minute per cycle followed by incubation in 100% ethanol for thirty seconds.
2. Air dried on Kim wipes then re-incubated in ethanol for about 30 s.
3. Rinsed three times in ddH_2_O for about one minute per cycle and assuring that the membrane remained immersed and fully wet.
4. Rinsed twice for about two minutes per cycle in Tris-buffered saline with 0.05% Tween 20 (TBST) followed by three, one minute rinses in ddH_2_O.
5. Incubated in a 10% ethanol, 7% acetic acid solution for fifteen minutes followed by four, 5-min rinses in ddH_2_O.
6. Incubated in Sypro Ruby Protein Blot Stain (Thermo-Fisher – s11791) for fifteen minutes followed by three, 1-min rinses in ddH_2_O.
7. Imaged with the ChemiDoc MP System (Bio-Rad) using the “Blots”, “Sypro Ruby”, “AutoExpose” settings.
8. Membranes were then washed four times in TBST for 5 min per cycle.

### Confirming transfer by membrane staining using no-stain protein labeling reagent (Thermo-Fisher—A44449)

Post transfer, the membranes underwent the following procedure:

1. Washed in ddH_2_O for at least 2 min post-transfer.
2. Incubated two membranes with shaking in 20 ml of No-Stain Protein Labeling Reagent (Thermo-Fisher – A44449) for about 10 min.
3. Washed for about 2 min up to three times in ddH_2_O with shaking.
4. Imaged with the ChemiDoc MP System (Bio-Rad) using the “Blots”, “Sypro Ruby”, “AutoExpose” settings.
5. Washed four times in TBST for five minutes per cycle.

### Membrane blocking and antibody incubations

Membranes were blocked for about one hour in a solution containing TBST and 5% BSA (blocking solution) with gentle shaking at room temperature. This was followed by incubation in 10 ml of diluted primary antibodies (Table 2) in blocking solution with gentle shaking either at 4 degrees overnight or 2.5 h at room temperature. The membranes were then washed four times for approximately five minutes with vigorous shaking at room temperature in TBST. This followed a one-hour incubation of each membrane with gentle shaking at room temperature with a mixture of the diluted secondary antibodies (Table 2) in blocking solution followed by four washes in TBST for approximately 5 min with vigorous shaking at room temperature.

### Antibody incubations

In order to delineate detection and quantification between the targets, the membranes were split for separate incubations with either goat-anti-rabbit HRP for detection of phospho-β-catenin or with goat-anti-mouse HRP for detection of β-catenin and α-tubulin (Table 2). HRP conjugated secondary antibodies were co-incubated with the fluorescent-labelled antibodies (Table 2) to permit detection of the same proteins by both fluorescence and chemiluminescence from the identical membranes to minimize technical variability.

### Blot imaging and analysis

The blots were first imaged using the multichannel feature of the ChemiDoc MP Imaging System (Bio-Rad) with “Blots”, “Alexa 647”, “Alexa 488” and “Colorimetric” applications using the auto expose settings. This was followed by chemiluminescence detection using the Clarity or Clarity Max Western ECL Substrate (Bio-Rad – 1705062) and the ChemiDoc MP Imaging System with “Blots”, “Chemiluminescence” application and auto expose or manual settings. All densitometric data were acquired and translated using Image Lab software (Bio-Rad) with data analysis, graphing and statistics using GraphPad Prism software.

### Simple membrane stripping protocol for multi-protein detection by chemiluminescence

Since the phosphorylated and total protein isoforms of β-Catenin resolve at the same molecular weight with SDS-PAGE, their individual signals cannot be distinguished with chemiluminescence that emits photons at a single wavelength. Hence, the two proteins would appear as a single band during detection. This necessitates sequential data capture by first detecting one isoform followed by the stripping of the associated antibodies from the membrane to enable incubation of the antibodies to the other isoform and subsequent detection. A simple stripping procedure was adopted whereby the membranes were incubated in a in a 10% ethanol, 7% acetic acid solution overnight at 4 degrees followed by four, 3-min rinses in ddH_2_O and three, 3-min washes in TBST. A similar protocol has been tested to reprobe western blot membranes^20^. Fresh primary and secondary antibodies were then applied according to the “Membrane Blocking and Antibody Incubations” procedure (above) with imaging using chemiluminescence according to the “Blot Imaging” section (above).

### Western blot data analysis workflow

Data analysis and associated calculations for relative quantification involves multiple steps as follows (Fig. 4 and supplemental WB Data Analysis EXCEL Spreadsheet template):

1. For experiments where the sample number exceeds the number of available wells per gel (which is usually the case) generate an “Interblot Control” (IBC) sample from a pool of positive samples. Assure an adequate quantity of Control to load on each gel of the experiment at an appropriate dilution to be in the linear range for each tested target. This may require dedicating two or three lanes to load a few dilutions of the IBC sample such that at least one dilution is well within the linear range for each target.
2. Post-transfer from gel to membrane, image the transferred total protein using a compatible, fluorescent total protein stain (see "Methods" section). These data are ultimately required for normalization but can also be immediately used to assess transfer efficiency and protein quality/uniformity between samples as a post-transfer quality control step (Fig. 3B).
3. After probing, washing and imaging the membrane, extract the background-subtracted raw intensity values for each target protein from the imaging system data file using software that does not manipulate the raw image data (Fig. 3B).
4. Normalize the data by calculating the ratio of the raw intensity per target to that of the corresponding total protein lane (i.e.: [Target Protein – Lane X]/[Total Protein – Lane X]). Choose the same region of the lanes within each gel that gives the best signal-to-noise (Fig. 4 – Column 5) (Fig. 3B).
5. Calculate the relative, normalized ratio for each sample within each gel/blot (Fig. 3B). There are two approaches that can be taken to achieve solid results:
  - Within each gel, divide the normalized ratio for the target(s)/sample(s) (see point iv) by that of the pooled IBC sample (see point i) loaded on each gel (i.e.: ([Target Protein − Lane X]/[Total Protein − Lane X])/([Target Protein − IBC]/[Total Protein − IBC])) (Fig. 4—Column 8).
  - The average normalized ratio of all samples within a given experimental group can be used for the relative ratio calculation. However, these samples must all be present on the same gel/blot which would only be the case for a small experiment. The calculation would be as follows: (i.e.: ([Target Protein − Lane X]/[Total Protein − Lane X])/([Average of [Normalized Target Proteins (see point iv) for Given Bio-Group])) (Fig. 4—Column 6).
6. To assess the relative, normalized expression differences between biological groups for the entire sample set spanning multiple blots, divide each relative, normalized sample/target (see step v) by the average relative, normalized expression for the replicate sample/targets in the assigned control biological group (Bio Group) (Fig. 3B). This will assure that the average expression of the control bio group is equal to one and those of the experimental bio groups will have a fold difference versus control (Fig. 4—Columns 7 or 9).
7. Assure that the relative, normalized data within bio group (step 6) is normally distributed (typically using a Shapiro–Wilk Test) and perform statistical analysis on the data using parametric or nonparametric tests depending on data normality.
8. Presuming the results are not normally distributed which can be the case with ratio metric derived results, the data can potentially be log transformed to achieve normality for parametric statistical testing.

## Supplementary Information

Supplementary Legends.

Supplementary Figure S1.

Supplementary Figure S2.

Supplementary Information 4.

## Data Availability

The datasets used and/or analysed during the current study available from the corresponding author on reasonable request.
