# Supplementary material for: A critical path to producing high quality, reproducible data from quantitative western blot experiments
Source: Sci Rep. 2022 Oct 20;12:17599. doi: 10.1038/s41598-022-22294-x (PMC9585080; doi:10.1038/s41598-022-22294-x)
Supplement: Supplementary file 1 — Supplementary Legends. [file 41598_2022_22294_MOESM1_ESM.docx]

**Supplementary Figure Legends**

**Supplemental Figure 1.** Full membrane images for cropped western blot data in Figure 1B. The cropped images from the fluorescent western blot in Figure 1B are presented. Directly below each cropped image for Alexa 488 and Alexa 647, the full blot image is shown. The full blot image for total protein transferred to the membrane is also displayed.

**Supplemental Figure 2.** Full membrane images for cropped western blot data in Figure 2. The cropped images from the chemiluminescent western blots in Figures 2A1 and 2A2 are presented. Directly below each cropped image, the full blot image is shown. The full blot image for total protein transferred to each membrane is also displayed.
