# Supplementary material for: A critical path to producing high quality, reproducible data from quantitative western blot experiments
Source: Sci Rep. 2022 Oct 20;12:17599. doi: 10.1038/s41598-022-22294-x (PMC9585080; doi:10.1038/s41598-022-22294-x)

Supplemental Figure 1
(Full Membrane Images for Cropped Data in Figure 1)

B) Cropped and full blot images

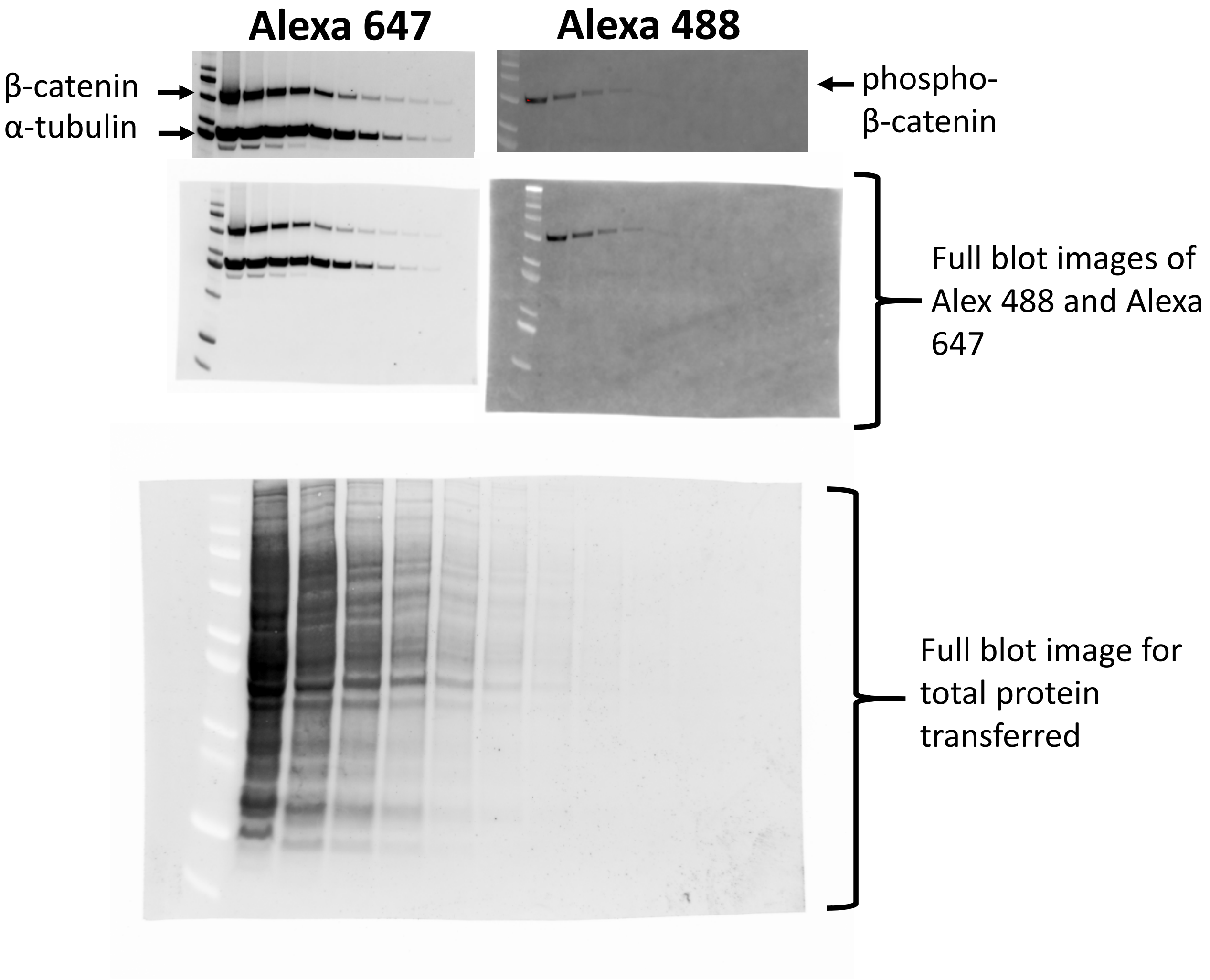

Supplement: Supplementary file 2 — Supplementary Figure S1. [file 41598_2022_22294_MOESM2_ESM.pdf]
