# Supplementary figures and images for: A critical path to producing high quality, reproducible data from quantitative western blot experiments
Source: Sci Rep. 2022 Oct 20;12:17599. doi: 10.1038/s41598-022-22294-x (PMC9585080; doi:10.1038/s41598-022-22294-x)

## Supplemental Figure 2 (Full Membrane Images for Cropped Data in Figure 2)

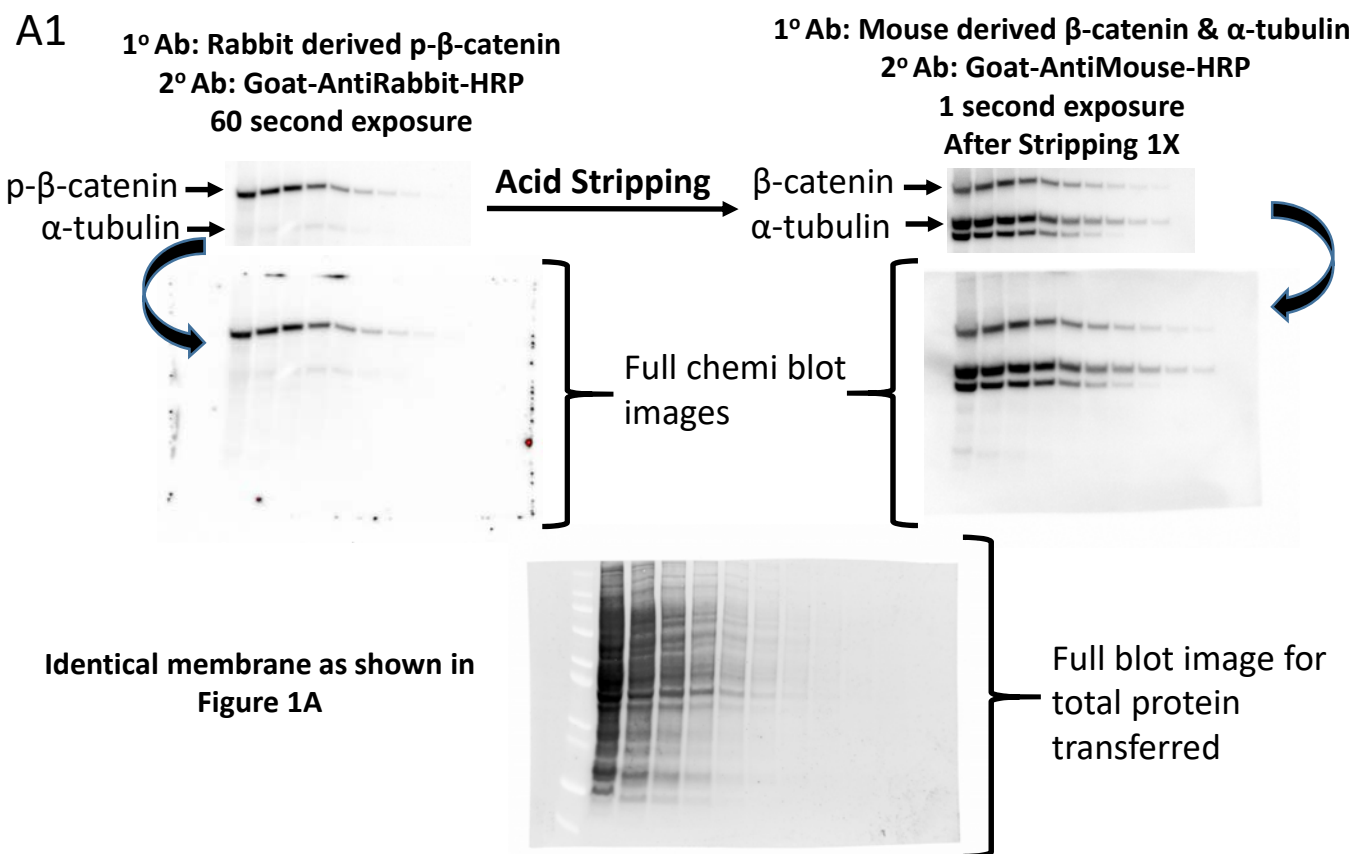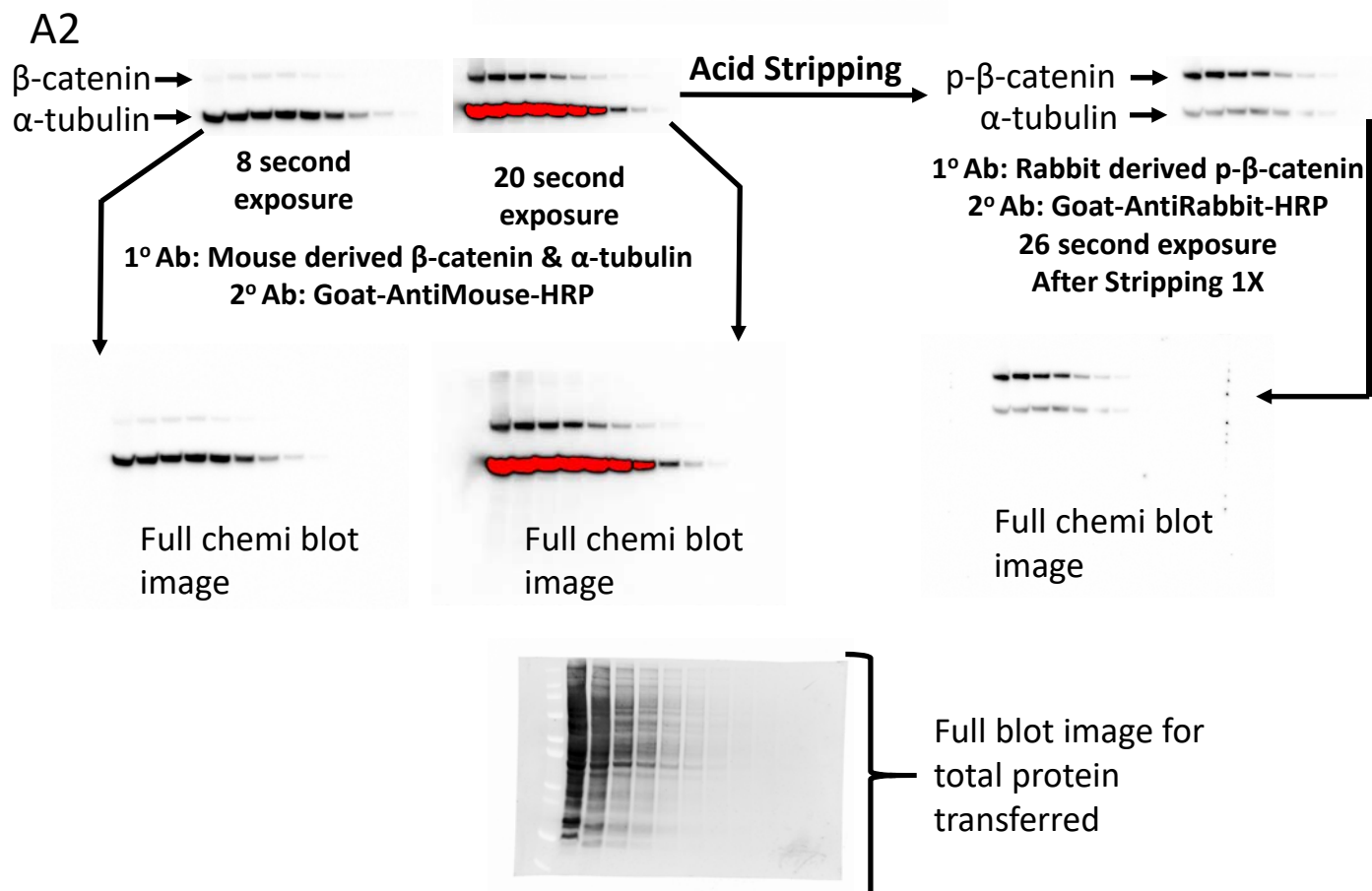

Supplement: Supplementary file 3 — Supplementary Figure S2. [file 41598_2022_22294_MOESM3_ESM.pdf]
